# Growth and Mycotoxin Production by *Chaetomium globosum* Is Favored in a Neutral pH

**DOI:** 10.3390/ijms9122357

**Published:** 2008-11-26

**Authors:** Matthew R. Fogle, David R. Douglas, Cynthia A. Jumper, David C. Straus

**Affiliations:** 1 Department of Microbiology and Immunology, Texas Tech University Health Sciences Center 3601 4th Street Mail stop 6591 Lubbock, TX 79430, USA; 2 Department of Internal Medicine, Texas Tech University Health Sciences Center 3601 4th Street Mail stop 6591 Lubbock, TX 79430, USA

**Keywords:** Ambient pH, chaetoglobosin, Chaetomium globosum, indoor air quality, mycotoxin

## Abstract

*Chaetomium globosum* is frequently isolated in water-damaged buildings and produces two mycotoxins called chaetoglobosins A and C when cultured on building material. In this study, the influence of ambient pH on the growth of *C. globosum* was examined on an artificial medium. This fungus was capable of growth on potato dextrose agar ranging in pH from 4.3 to 9.4 with optimal growth and chaetoglobosin C production occurring at a neutral pH. In addition, our results show that sporulation is favored in an acidic environment.

## 1. Introduction

*Chaetomium globosum* is a fungus frequently isolated in water-damaged buildings [1–3]. When cultured on building material, *C. globosum* produces chaetoglobosins A and C [4]. The presence of these mycotoxins can be lethal to mammalian cells which act by binding to actin leading to inhibition of cell division, locomotion, and formation of cell surface projections [5, 6]. Injection of chaetoglobosin A in rodents was shown to be fatal at relatively low doses [7]. Due to its high frequency of isolation in buildings and toxicity of its metabolites, we felt *C. globosum* warrants further study.

In a previous study, we examined the growth of *C. globosum* on four commercially available media. We found that the medium that supported the best growth also supported the highest production of chaetoglobosins A and C [3]. Based on these results, we hypothesized that mycotoxin production is directly related to growth. In this study, the influence of ambient pH on the growth of *C. globosum*, as well as the sporulation and production of chaetoglobosins A and C, was examined on an artificial medium. We project that as growth is reduced under sub-optimal conditions, mycotoxin production will also decline.

## 2. Results and Discussion

On the day of inoculation, the pH of sterile unbuffered potato dextrose agar (PDA) was 5.63 while the pH of sterile buffered PDA ranged from 3.51 to 9.35. After four weeks of incubation at room temperature (25 °C), the pH of the sterile agar dropped (as much as 0.19) for the unbuffered, Tris buffered and Tris-maleate buffered PDA, and increased (up to 0.24) on the citrate-phosphate buffered and carbonate-bicarbonate buffered PDA (Table 1).

**Figure 1.**
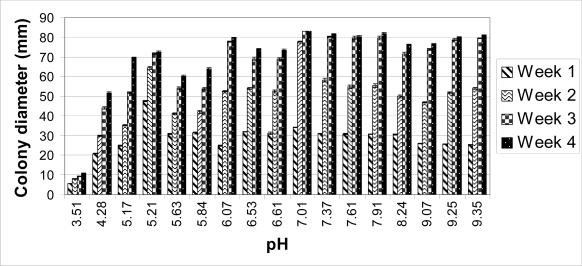
Comparison of colony diameters of *C. globosum* on buffered and unbuffered potato dextrose agar. The actual pH of each medium on the day of inoculation is listed on the x-axis. Colony diameters were measured every week. The maximum diameter of each plate was 83 mm. Mean and standard error of the mean are shown (n = 15 plates).

**Figure 2.**
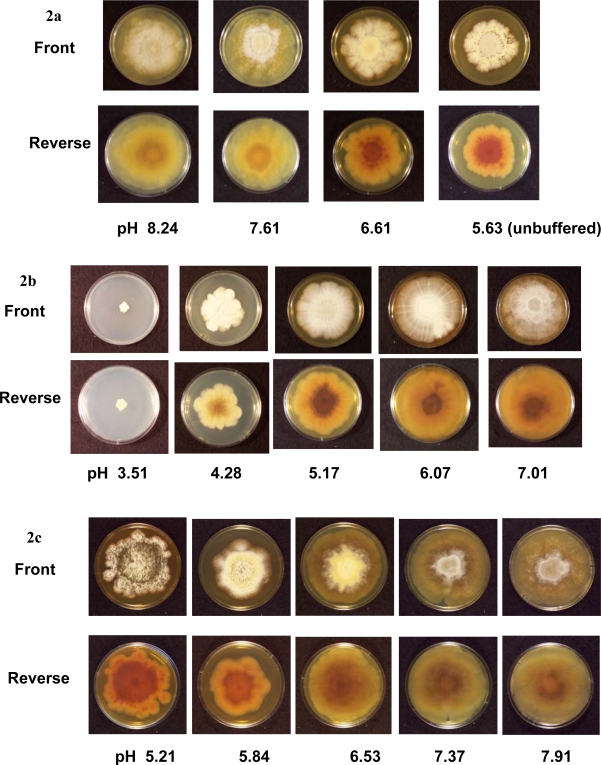
Photographs of *C. globosum* colonies at 4 weeks on Tris buffered and unbuffered potato dextrose agar (a), on citrate-phosphate buffered potato dextrose agar (b) and on Tris-maleate buffered potato dextrose agar (c). The center of each agar plate was inoculated with 500 *C. globosum* spores suspended in 20 μL of water. These photographs depict the front and reverse sides of agar plates with *C. globosum* colonies after four weeks of incubation at room temperature.

The colonies on unbuffered PDA (pH 5.63) reached 60 mm in diameter after four weeks. When the pH of the PDA was raised with the Tris buffer (pH 6.61, 7.61 and 8.24), the average colony sizes were higher compared to those on unbuffered PDA (Figures 1 and 2A). Perithecia were present on unbuffered PDA and all Tris buffered media by four weeks. Ascospores were observed after four weeks on unbuffered PDA and Tris buffered PDA at pH 6.61 and 7.61, but not at pH 8.24. No ascospores were observed up to eight weeks post-inoculation on Tris buffered PDA at pH 8.24 (Table 2).

A citrate phosphate buffer was used to obtain PDA ranging in pHs from 3.51 to 7.01 (Table 1). The colonies cultured at a pH of 7.01 covered the entire plate (83 mm in diameter) four weeks post-inoculation (Figure 2B). As pH decreased on each medium, colony sizes decreased. After four weeks, the colonies grown at a pH of 3.51 only reached an average of 11 mm in diameter (Figure 1) and no hyphal filaments were observed on the tape slides (data not shown). At a pH of 4.28, 5.17, 6.07 and 7.01, no perithecia were produced four weeks post-inoculation, but did eventually form eight weeks post-inoculation. After eight weeks, ascospores were present at a pH of 4.28, 5.17 and 7.01 (Table 2).

The carbonate-bicarbonate buffer raised the pH of PDA higher than the Tris buffer (pH 9.07, 9.25 and 9.35) (Table 1). The average colony size on each carbonate-bicarbonate buffered medium was lower compared to the citrate-phosphate buffered PDA at a pH of 7.01 (Figure 1). No perithecia or ascospores were observed at 4, 6 or 8 weeks post-inoculation (Table 2).

Tris-maleate buffer resulted in PDA ranging in pH from 5.21 to 7.91 (Table 1). After two weeks, the largest colonies were observed at the lowest pH. At three and four weeks, the colonies with the largest diameter were on PDA with a pH of 7.37 and 7.91 (Figures 1 and 2C). At four weeks, numerous ascospores were observed at a pH of 5.21 and 5.84, while few or no ascospores were seen on the other Tris-maleate buffered PDA (data not shown). Within six weeks, ascospores were produced on each medium (Table 2).

**Figure 3.**
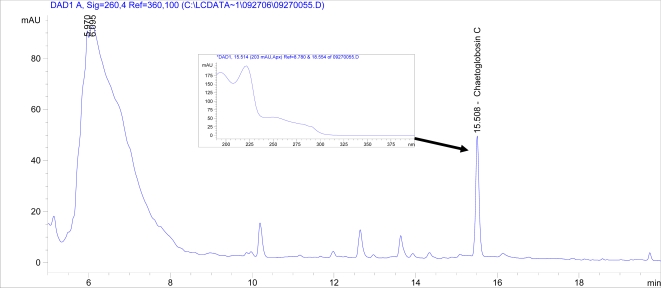
HPLC chromatogram with UV spectra of selected peak inserted. The chromatogram shows the signal obtained from the methanol extract of *C. globosum* grown on five citrate phosphate buffered potato dextrose agar plates at a pH of 7.01 for four weeks. The retention times (min) are plotted on the x-axis and the peak sizes (in milli-absorbance units) on the y-axis. For UV spectrum (inset), wavelengths (in nanometers) are plotted on the x-axis and peak sizes (in milli-absorbance units) on the y-axis.

Overall, the largest colonies were obtained at a neutral pH (7.01) (Figure 1). By two weeks, these colonies were significantly larger compared to every other medium. By four weeks, the average colony size for each medium was significantly smaller except at a pH of 6.07, 7.37, 7.61, and 7.91 compared to a pH of 7.01 (data not shown). The total number of spores for each group was determined as previously described [3]. Detectable levels of ascospores were observed on the following three out of seventeen media at four weeks post-inoculation: 4,240,000 spores per group (i.e., five agar plates) on unbuffered PDA (pH 5.63); 13,500,000 and 2,960,000 spores per group on Tris-maleate buffered PDA, pH 5.21 and 6.53, respectively. Tape slides revealed the production of ascospores on four other buffered media (Tris buffered PDA at pH of 6.61 and 7.61; Tris-maleate buffered PDA at pH of 8.84 and 7.91) (Table 2), which was below the detection limit of the hemacytometer (10,000 spores/mL). The production of chaetoglobosins A and C were evaluated as previously described using HPLC [3]. Chaetoglobosin C was detected at a pH of 7.01 (an average of 203 μg per five agar plates), but not from media at any other pH (Figure 3). No chaetoglobosin A was detected in any of the samples (data not shown).

Few studies have examined the influence of ambient pH on the growth of *C. globosum*. The optimal pH range for the growth of *C. globosum* was previously described as 7.1 to 10.4 [8]. Our results indicate this fungus could grow over a range of different pH values (approximately 4.3 to 9.4). Although *C. globosum* grew at a pH of 3.51, these colonies were small in size and had an abnormal morphology (Figure 2). The growth of *C. globosum* is optimal at a neutral pH (Figure 1).

Detectable levels of chaetoglobosin C were only observed on the medium with the largest *C. globosum* colonies. This finding is consistent with our results from a previous study [3] which suggest that the production of chaetoglobosins is directly related to growth. After examining the growth of *C. globosum* on four commercially available media, we found that the medium that supported the best growth also supported the highest production of chaetoglobosins A and C [3].

Ambient pH has been shown to influence metabolite production in other filamentous fungi. The best studied regulatory system is in *Aspergillus nidulans* which is controlled by a transcription factor called PacC [9]. Under alkaline conditions, PacC activates alkaline-expressed genes such as *acvA* and *ipnA* which are involved in penicillin synthesis and represses acid-expressed genes such as *stcU* which is involved in sterigmatocystin synthesis. Other filamentous fungi with PacC homologs include *Aspergillus niger*, *Aspergillus oryzae*, *Penicillium chrysogenum*, *Acremonium chrysogenum*, *Sclerotinia sclerotiorum* [9], and *Fusarium verticillioides* [10]. A hypothetical protein similar to PacC has been located within the *C. globosum* genome [11]. Assuming this fungus has a similar regulatory system as in *A. nidulans*, these results suggest that chaetoglobosin production is not under its control.

The formation of perithecia and ascospores by *C. globosum* appears to be favored in an acidic environment and inhibited under basic conditions on an artificial medium. After four weeks, ascospores were present in detectable levels on unbuffered PDA (pH 5.63) and Tris-maleate buffered PDA (pH 5.21 and 6.53) (Table 2). *C. globosum* eventually produced perthecia and ascospores on citrate-phosphate buffered PDA eight weeks post-inoculation. No ascospores were produced on Tris buffered PDA at pH 8.24 or on the carbonate-bicarbonate buffered PDA at pH 9.07, 9.25 and 9.35 (Table 2). It is also possible that this inhibition of sporulation at a basic pH is due to the presence of one of the buffer’s components, although the mechanism remains unknown at the present time.

## 3. Experimental Section

*Chaetomium globosum* American Type Culture Collection 16021 (ATCC, Manassas, Virginia) was cultured on Difco potato dextrose agar (PDA) (Becton, Dickinson and Company, Sparks, Maryland) at room temperature (RT or 25 °C) until confluent growth and sporulation were achieved as previously described [3].Double-strength PDA was mixed with an equal volume of each buffer to obtain the desired concentration of medium as described by Kim *et al*. [13]. The following buffers were prepared at the predicted pH as described by Gomori (1955): citrate-phosphate buffer, pH 3.0, 4.0, 5.0, 6.0 and 7.0; Tris (hydroxymethyl) aminomethane (Tris) buffer, pH 7.2, 8.0, and 9.0; carbonate-bicarbonate buffer, pH 9.2, 10.0 and 10.7; and Tris (hydroxymethyl) aminomethane-maleate (Tris-maleate) buffer, pH 5.2, 6.0, 7.0, 8.0, and 8.6 [12]. The buffers and PDA were autoclaved separately and aseptically mixed during cooling. The buffered medium was poured into Petri dishes (VWR International, Inc., Aurora, Colorado) and allowed to solidify at RT.

Every week, colony diameters were measured at right angles on each agar plate resulting in two readings. Significance was determined using Kruskal-Wallis analysis of variance on ranks (P<0.05) followed by Dunnett’s post-hoc analysis to determine differences in colony diameter using the SigmaStat 2.0 software (Systat Software Inc., Richmond, California).

The pH of agar plates was determined by removing a 1 inch circular piece of agar from the center of each agar plate and placing it in a 50 mL polypropylene tube (VWR International Inc., Aurora, Colorado). Twenty-five mL of water were added to each tube and the agar was allowed to incubate at RT for 1 h. After removing the agar, the pH of the water was measured with a Model 15 pH meter (Fisher Scientific, Pittsburg, Pennsylvania) while stirring.

For each buffered medium, tape slides were taken from a representative plate to examine sporulation at four, six and eight weeks post-inoculation. Clear adhesive tape was used to sample fungal growth and placed onto a glass slide containing lactophenol cotton blue [14]. Slides were examined with a BH-2 transmitted light microscope (Olympus, Center Valley, Pennsylvania) at a magnification of 100x or 400x.

After four weeks, the agar plates were placed into groups of five and soaked in 250 mL of methanol to extract chaetoglobosins A and C. The methanol extracts were then passed through a fiberglass filter (GF/D 1823, Whatman, Clifton, NJ) to remove the large particulates. The extracts were transferred to 1 L beakers and allowed to dry under a fume hood at RT.

The dried contents of each 1 L beaker were dissolved in methanol (20 mL) and passed through a 0.45 μm glass microfilter (Autovial GMF, Whatman, Clifton, NJ) into a 20 mL glass scintillation vial. The vials were placed under a fume hood until dry. This process was repeated twice (for a total of three times) to recover any residual material left in the beaker.

After allowing to completely dry, each scintillation vial received 2 mL of methanol. The vials were vortexed until the residue was dissolved. The extracts were passed through 0.2 μm syringe filters (25 mm sterile nylon membrane, Fisherbrand, Pittsburg, PA) into 2 mL glass vials (C4000-1W, National Scientific Company, Rockwood, TN).

Detection of chaetoglobosins A and C was performed using an 1100 Series HPLC system (Agilent Technologies, Palo Alto, CA) equipped with a UV-visible diode array detector. An Agilent Eclipse C8 analytical column (400 mm [250 plus 150 mm] by 4.6 mm; particle size, 5 μm) and a 12.5 mm guard column set at 40 °C were used in the analyses. The flow rate was set at 1.0 mL/min. Crude toxin samples in methanol were run in a mobile phase in which the gradient changed from a 35% solution of 95% water/ 5% acetronitrile to 80% acetonitrile in 20 minutes. Samples were read at 260 nm and were analyzed using Chemstation software (Agilent Technologies, Palo Alto, CA).

## 4. Conclusions

*C. globosum* was capable of growth on potato dextrose agar ranging in pH from 4.3 to 9.4. The optimal growth of this fungus, as well as chaetoglobosin C production, occurred at a neutral pH. In addition, our results show that sporulation by *C. globosum* is favored in an acidic environment.

## Figures and Tables

**Table 1.** pH measurements of sterile potato dextrose agar.

Medium	Predicted pH of Buffer	Actual pH^a^	
		Sterile Agar	
		Day 0	Day 28
Unbuffered PDA	n/a	5.63	5.47
Tris buffered PDA	7.20	6.61	6.50
	8.00	7.61	7.59
	9.00	8.24	8.19
Citrate-phosphate buffered PDA	3.00	3.51	3.69
	4.00	4.28	4.49
	5.00	5.17	5.40
	6.00	6.07	6.31
	7.00	7.01	7.23
Carbonate-bicarbonate buffered PDA	9.20	9.07	9.13
	10.00	9.25	9.26
	10.70	9.35	9.39
Tris-maleate buffered PDA	5.20	5.21	5.02
	6.00	5.84	5.75
	7.00	6.53	6.45
	8.00	7.37	7.30
	8.60	7.91	7.83

^a^ Average of three samples is shown.

**Table 2:** Effect of pH on the sporulation.

Medium	Predicted pH of buffer	Tape slide results					
		Presence of perithecia			Presence of ascospores		
		Week 4	Week 6	Week 8	Week 4	Week 6	Week 8
Unbuffered PDA	n/a	+	+	+	+	+	+
Tris buffered PDA	7.2	+	+	+	+	+	+
	8.0	+	+	+	+	+	+
	9.0	+	+	+	−	−	−
Citrate-phosphate buffered PDA	3.0	−	NT	NT	−	NT	NT
	4.0	−	+	+	−	−	+
	5.0	−	+	+	−	−	+
	6.0	−	−	+	−	−	−
	7.0	−	+	+	−	−	+
Carbonate-bicarbonate buffered PDA	9.2	−	−	−	−	−	−
	10.0	−	−	−	−	−	−
	10.7	−	−	−	−	−	−
Tris-maleate buffered PDA	5.2	−	−	−	+	+	+
	6.0	+	+	+	+	+	+
	7.0	+	+	+	+	+	+
	8.0	+	+	+	−	+	+
	8.6	+	+	+	+	+	+

^a^ Tape slides were taken from a single agar plate at four, six or eight weeks post-inoculation. Presence or absence of perithecia and ascospores is indicated with a “+” or “−” respectively. Samples not taken are indicated by “NT”.
